# Associations of metal exposure with hyperuricemia and gout in general adults

**DOI:** 10.3389/fendo.2022.1052784

**Published:** 2022-12-02

**Authors:** Jing Xu, Xu Zhu, Rutai Hui, Yujie Xing, Junkui Wang, Shuang Shi, Yong Zhang, Ling Zhu

**Affiliations:** ^1^ Department of Cardiology, Shaanxi Provincial People’s Hospital, Xi’an, Shaanxi, China; ^2^ Department of Cardiology, The First Affiliated Hospital of Nanjing Medical University, Jiangsu Province Hospital, Nanjing, China; ^3^ Department of Cardiology, State Key Laboratory of Cardiovascular Disease, Fuwai Hospital, National Center for Cardiovascular Diseases, Chinese Academy of Medical Sciences and Peking Union Medical College, Beijing, China; ^4^ Department of Cardiology, The Third Affiliated Hospital of Xi’an Jiaotong University, Xi’an, Shaanxi, China

**Keywords:** blood metals, uric acid, hyperuricemia, gout, weighted quantile sum

## Abstract

**Background:**

Epidemiological evidence of the associations between metal exposure and gout-related outcomes (including serum uric acid [SUA], hyperuricemia and gout) is scarce. The aim of the study is to investigate the associations of metal exposure with SUA, hyperuricemia and gout in general adults.

**Methods:**

In this study, the exposure to five blood metals (mercury, manganese, lead, cadmium and selenium) of general adults was analyzed based on the National Health and Nutrition Examination Survey (NHANES) from 2011 to 2018 (n = 14,871). Linear, logistic and weighted quantile sum (WQS) regression models were applied to examine the associations of blood metals with gout-related outcomes. Possible dose-response relationships were analyzed through restricted cubic spline regression.

**Results:**

Compared with the lowest quartile of blood metals, mercury (quartile 2 and 4), lead (quartile 2, 3, and 4) and selenium (quartile 2 and 4) were found to be positively correlated with SUA and hyperuricemia. Higher levels of mercury and lead were associated with gout, but only those in the fourth quartile had statistical significance (OR [95%CI]: 1.39 [1.10-1.75] and 1.905 [1.41-2.57]) respectively). The WQS index of the blood metals was independently correlated with SUA (β [95%CI]: 0.17 [0.13-0.20]), hyperuricemia (OR [95%CI]: 1.29 [1.16-1.42]) and gout (OR [95%CI]: 1.35 [1.15-1.58]). Among them, lead was the most heavily weighted component (weight = 0.589 for SUA, 0.482 for hyperuricemia, and 0.527 for gout). In addition, restricted cubic spline regression models showed a linear association of lead with the prevalence of hyperuricemia and gout.

**Conclusion:**

Our results suggested that blood metal mixtures were positively associated with gout-related outcomes, with the greatest effect coming from lead.

## Introduction

Uric acid is an end product of the metabolism of purine analogues. If there is an excessive production (an increased endogenous purine catabolism and an excessive exogenous purine intake) or an impaired excretion of uric acid, it may lead to an increase in the level of serum uric acid (SUA) (1). Hyperuricemia, which is defined when the level of SUA exceeds a certain range (2), is a chronic metabolic disease in which large amounts of uric acid are deposited in joints and tissues for a long time, eventually developing into gout (3, 4). Patients with severe gout may experience recurrent acute attacks with subsequent joint damage and bone erosion, leading to functional impairments and the combination of other chronic diseases such as gouty nephropathy, cardiovascular diseases and diabetes (5–8).

Scholars have conducted significant research on the factors that influence hyperuricemia, including basic demographic features such as age and gender, lifestyle factors like smoking and alcohol intake, and chronic disorders such as hypertension, diabetes mellitus, and dyslipidemia. In addition, there is a growing interest in the possibility of being exposed to environmental heavy metals (9). Metallic elements enter the human body through multiple pathways and cause significant harm to human health (10). Inconsistent results have been obtained from studies examining the association between heavy metals and hyperuricemia. In the majority of studies, the association between one or more heavy metals and hyperuricemia has been investigated without taking into account the combined effects of metal combinations on the human body (11–13). Since the level of metals in the blood is a valuable indicator of the level of human exposure to metals, it is crucial to investigate the association between the level of mixed metals in blood and the prevalence of hyperuricemia.

In this study, based on the data from the National Health and Nutrition Examination Survey (NHANES) 2011–2018 on general adults, we assessed the relationship of five metals (mercury, manganese, lead, cadmium and selenium) and their mixtures in blood with gout-related outcomes (including SUA, hyperuricemia and gout) through linear, logistic and weighted quantile sum (WQS) regression models. These associations were evaluated to provide epidemiological evidence for further studies on the correlation of metal exposure with hyperuricemia and gout.

## Materials and methods

### Study population

The NHANES is a nationwide cross-sectional survey conducted periodically by the National Center for Health Statistics (NCHS) of Centers Disease Control and Prevention (CDC), which aims to assess the health and nutritional status of adults and children in the United States. The research protocols were approved by the National Center for Health Statistics (NCHS) Research Ethics Review Board, and all participants provided written informed consent.

All interview and medical examination data can be downloaded from the official website (https://www.cdc.gov/nchs/nhanes), from which we obtained the data over four cycle years (namely 2011–2012, 2013–2014, 2015–2016 and 2017–2018). Participants with missing data on five blood metals (n=14,150) were excluded. Moreover, participants aged <18 with missing information on assessment data of gout (n=9,651) or SUA (n=335) and those who were pregnant (n=149) were ruled out. Lastly, a total of 14,871 participants were included in the analysis **(** **Figure S1** **)**.

### Assessment of metal levels in blood

In NHANES 2011-2018, the available data for heavy metals detected in blood samples were mercury, manganese, lead, cadmium and selenium. Heavy metals in blood were measured directly by NCHS/CDC, which mainly used inductively coupled plasma dynamic reaction cell mass spectrometry (ICP-DRC-MS) to analyze the level of metals in participants. We investigated five metals, namely mercury, manganese, lead, cadmium and selenium, meanwhile analyzing their concentration and distribution in blood. The lower limit of detection (LOD) for blood mercury, manganese, lead, cadmium and selenium was 0.28μg/dL, 0.99μg/dL, 0.07μg/dL, 0.10μg/dL and 24.48 μg/dL respectively. The values that were less than the LOD were substituted with a lower LOD divided by the square root of 2.

### Assessment of SUA, hyperuricemia and gout

Serum samples were collected from participants and stored at –30°C until they were transported to NCHS/CDC for SUA testing. Hyperuricemia was defined as a level of SUA that was ≥416 μmol/L (7 mg/dL) in men and ≥357 μmol/L (6 mg/dL) in women [9]. Gout was defined as a physician’s self-reported diagnosis, obtained by asking the following question (“Has a doctor or other health professional ever told you that you have gout?”).

### Covariates

Information on baseline data was collected through questionnaires and laboratory tests, including age (years), gender (male or female), education level (below high school, high school and above high school), race/ethnicity (Mexican American, other Hispanic, non-Hispanic white, non-Hispanic black and other races), sedentary time (<3, 3-6, >6 hrs), body mass index (BMI, kg/m^2^), high-density lipoprotein cholesterol (HDL-C, mg/dL), total cholesterol (mg/dL), urinary creatinine (mg/dL), urinary albumin (ug/mL) and estimated glomerular filtration rate (eGFR, ml/min/1.73 m^2^). Poverty was assessed using the poverty income ratio (PIR) and defined according to a PIR cutoff that was <1 for a given family. Participants with a serum cotinine value that was >14 ng/mL were defined as smokers. Individuals consuming at least 12 alcoholic drinks in a single calendar year were defined as alcohol users. The intake of energy, nutrients and other food components was calculated by averaging the two values from two 24-hour recalled interviews. We obtained information on the prevalence of hypertension and diabetes among participants using self-reported questionnaires. In addition, the usage of anti-gout medicines, diuretics, and beta-blockers was recorded.

### Statistical analysis

The continuous variables were presented as the mean (standard deviation [SD]) or the median (interquartile range), and the categorical variables were presented as n (%). The Kolmogorov-Smirnov statistical test was used to determine if continuous variables were normally distributed. To normalize their distribution, the blood metals were log-transformed. Spearman’s correlation was utilized to determine the exposure correlation coefficients for all metals.

Using multiple linear regression models, corrected β-coefficients and 95% confidence intervals (CIs) were calculated to determine the level of SUA associated with the blood metals. Multiple logistic regression models were used to calculate adjusted odd ratios (ORs) and 95% CIs to assess the prevalence rate of hyperuricemia and gout associated with the blood metals. The blood metals were divided into quartiles, with the lowest quartile being considered the reference category. To investigate the dose-response curves of exposure variables and the prevalence of hyperuricemia and gout, a restricted cubic spline regression model with knots at the 5^th^, 35^th^, 65^th^, and 95^th^ percentile of each exposure variable was utilized.

The WQS regression model was used to assess the confounding effect of multiple exposure variables on a given dependent variable, each of which was given a weight in the model to indicate the magnitude of its effect on the dependent variable. In the model, 40% of the data was assigned to the training set and 60% to the validation set, meanwhile the training set was bootstrapped 1000 times to maximize the likelihood function of the linear model.

Finally, we conducted a stratified analysis in age (>50 years versus ≤ 50 years) and sex (male versus female). All models were adjusted by age, sex, education level, race, poverty, smoker, alcohol user, energy intake level, sedentary time, BMI, total cholesterol, HDL-C, eGFR, urinary creatinine, urinary albumin, diuretics, beta-blockers, diabetes and hypertension. We performed all statistical analyses using R software (Version 4.2.0).

## Results

### Population characteristics of participants

This study comprised 14,871 adults, whose baseline characteristics are presented in **Table 1**. The average age of the participants was 50.02 years, 49.3% of them were male, and 55.7% of them had completed education beyond high school. White non-Hispanics constituted 37.2% of the entire study population. The median level of SUA in the overall population was 5.30 (IQR 4.40, 6.40) mg/dL, and the prevalence of hyperuricemia as well as gout was 18.6% and 4.9%, respectively.

**Table 1:** Characteristics of the study population.

Variable	Overall (n= 14,871)
Age, years	50.02 (17.57)
Male, %	7328 (49.3)
Education level, %	
Below high school	3259 (21.9)
High school	3330 (22.4)
Above high school	8282 (55.7)
Race/ethnicity, %	
Mexican American	1953 (13.1)
Other Hispanic	1541 (10.4)
Non-Hispanic White	5527 (37.2)
Non-Hispanic Black	3342 (22.5)
Other race	2508 (16.9)
Poverty, %	3273 (22.0)
Smoker, %	3482 (23.4)
Alcohol user, %	10641 (71.6)
Sedentary time, h	
<3 h	2119 (14.2)
3-6 h	6868 (46.2)
>6 h	5884 (39.6)
Energy intake, kcal/day	1959.00 [1470.00, 2569.00]
Body mass index, kg/m^2^	3.09 (0.85)
HDL-C, mg/dL	1.37 (0.41)
Total cholesterol, mg/dL	190.29 (41.06)
Urinary creatinine, mg/dL	109.00 [61.00, 166.00]
Urinary albumin, µg/mL	8.50 [4.30, 18.50]
eGFR, ml/min/1.73 m^2^	93.62 (23.95)
Hypertension, %	5529 (37.2)
Diabetes, %	2107 (14.2)
Anti-gout medications, %	245 (1.6)
Diuretics, %	1828 (12.3)
Beta-blockers, %	1759 (11.8)
Uric acid, mg/dL	5.30 [4.40, 6.40]
Hyperuricemia, %	2766 (18.6)
Gout, %	736 (4.9)

Data are presented as median (IQR) or n (%); IQR, interquartile range; HDL-C, high-density lipoprotein cholesterol; eGFR, estimated glomerular filtration rate.

### Distribution and correlation of metals in blood

The concentration and detection rate of the blood metals are listed in **Table S1**. Mercury, manganese, lead, cadmium, and selenium all had median values of 0.78 ug/L, 9.26 ug/L, 1.02 ug/L, 0.32 ug/L, and 191.51 ug/L, respectively. The detection rate for lead was 99.9%, whereas all other blood metals were 100%. No notable correlations were discovered among the blood metals **(** **Figure S2** **)**, with the exception of a rather minor link between lead and cadmium (r = 0.34).

### Association between the blood metals and SUA

We used linear regression to assess the relationship between blood metals and SUA. The histogram of residuals showed that the residuals were normally distributed (**Figure S3**). Mercury, lead, and selenium were positively correlated with SUA (*P* < 0.05), as shown in **Table 2** of the findings of the linear regression analysis. In the model adjusted by all covariates, the β (quartile, 95% CI) of SUA was 0.084 (quartile 2, 0.032-0.136), 0.100 (quartile 3, 0.046-0.153) and 0.188 (quartile 4, 0.133-0.244) for mercury, 0.111 (quartile 2, 0.058-0.166), 0.193 (quartile 3, 0.135-0.250) and 0.287 (quartile 4, 0.225-0.350) for lead, and 0.082 (quartile 2, 0.030-0.135), 0.057 (quartile 3, 0.005-0.110) as well as 0.114 (quartile 4, 0.061-0.168) for selenium.

**Table 2:** Multiple linear regression associations of blood metal levels with serum uric acid in adults.

Metals	Continuous log2-transformed Metals	Quartile 1	Quartile 2	Quartile 3	Quartile 4	*P* for trend
		β	β (95%CI)	β (95%CI)	β (95%CI)	
Mercury, µg/L	0.046 (0.032, 0.059) ^***^	0.00 [Reference]	0.084 (0.032, 0.136) ^**^	0.100 (0.046, 0.153) ^***^	0.188 (0.133, 0.244) ^***^	<0.001
Manganese, µg/L	0.009 (-0.031, 0.048)	0.00 [Reference]	-0.016 (-0.069, 0.036)	0.002 (-0.051, 0.056)	0.020 (-0.036, 0.076)	0.394
Lead, µg/dL	0.107 (0.085, 0.129) ^***^	0.00 [Reference]	0.111 (0.058, 0.166) ^***^	0.193 (0.135, 0.250) ^***^	0.287 (0.225, 0.350) ^***^	<0.001
Cadmium, µg/L	-0.002 (-0.023, 0.019)	0.00 [Reference]	-0.029 (-0.082, 0.024)	-0.053 (-0.110, 0.005)	0.015 (-0.053, 0.083)	0.889
Selenium, µg/L	0.119 (0.020, 0.218) ^*^	0.00 [Reference]	0.082 (0.030, 0.135) ^**^	0.057 (0.005, 0.110) ^**^	0.114 (0.061, 0.168) ^***^	<0.001

Model was adjusted as age, sex, education level, race, poverty, smoker, alcohol user, energy intake levels, sedentary time, BMI, total cholesterol, high-density lipoprotein cholesterol, eGFR, urinary creatinine, urinary albumin, diuretics, anti-gout medications, beta-blockers, diabetes, and hypertension.

CI, confidence interval; ^*^ p < 0.05, ^**^ p < 0.01 and ^***^ p < 0.001.

### Association between the blood metals and hyperuricemia

**Figure 1:**
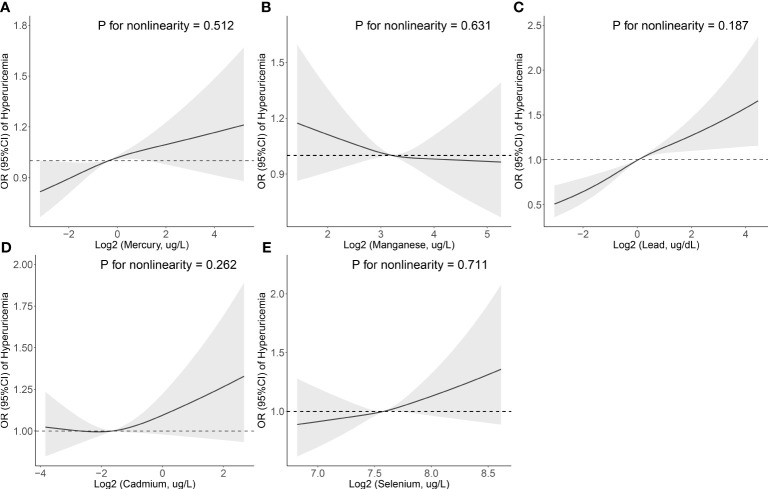
Association between the blood metal (**A**: Mercury, **B**: Manganese, **C**: Lead, **D**: Cadmium, and **E**: Selenium) levels and the prevalence of hyperuricemia. The odds ratio of hyperuricemia from a restricted cubic spline logistic regression model with knots at the 10^th^, 50^th^, and 90^th^ percentiles. Model was adjusted for age, sex, education level, race, poverty, smoker, alcohol user, energy intake levels, sedentary time, BMI, total cholesterol, high-density lipoprotein cholesterol, eGFR, urinary creatinine, urinary albumin, diuretics, beta-blockers, diabetes, and hypertension.

In multiple logistic regression analysis (**Table 3**), after the adjustment of all covariates, the OR (quartile, 95% CI) of hyperuricemia was 1.205 (quartile 2, 1.059-1.370) and 1.273 (quartile 4, 1.110-1.459) for mercury, 1.177 (quartile 2, 1.022-1.356), 1.267 (quartile 3, 1.094-1.468) and 1.538 (quartile 4, 1.316-1.798) for lead, and 1.155 (quartile 2, 1.013-1.317) as well as 1.239 (quartile 4, 1.086-1.414) for selenium. In addition, the restricted cubic spline revealed a positive linear association between three blood metals (mercury, lead, and selenium) and the prevalence of hyperuricemia (*P* for nonlinearity = 0.512, 0.187 and 0.711) (**Figure 1**).

**Table 3:** Multiple logistic regression associations of blood metal levels with hyperuricemia in adults.

Metals	Continuous log2-transformed Metals	Quartile 1	Quartile 2	Quartile 3	Quartile 4	*P* for trend
		OR	OR (95%CI)	OR (95%CI)	OR (95%CI)	
Mercury, ug/L	1.05 (1.02-1.09) ^**^	1.00 [Reference]	1.21 (1.06-1.37) ^**^	1.05 (0.92-1.20)	1.27 (1.11-1.46) ^**^	0.001
Manganese, ug/L	0.95 (0.86-1.04)	1.00 [Reference]	0.92 (0.81-1.05)	0.94 (0.82-1.07)	0.96 (0.84-1.11)	0.621
Lead, ug/dL	1.17 (1.11-1.23) ^***^	1.00 [Reference]	1.18 (1.02-1.36) ^*^	1.27 (1.09-1.47) ^**^	1.54 (1.32-1.80) ^***^	<0.001
Cadmium, ug/L	1.03 (0.98-1.08)	1.00 [Reference]	1.07 (0.94-1.21)	1.00 (0.87-1.15)	1.15 (0.97-1.35)	0.224
Selenium, ug/L	1.26 (0.99-1.61)	1.00 [Reference]	1.16 (1.01-1.32) ^*^	1.05 (0.92-1.20)	1.24 (1.09-1.41) ^***^	0.006

Model was adjusted as age, sex, education level, race, poverty, smoker, alcohol user, energy intake levels, sedentary time, BMI, total cholesterol, high-density lipoprotein cholesterol, eGFR, urinary creatinine, urinary albumin, diuretics, anti-gout medications, beta-blockers, diabetes, and hypertension.

CI, confidence interval; ^*^ p < 0.05, ^**^ p < 0.01 and ^***^ p < 0.001.

### Association between the blood metals and gout

**Figure 2:**
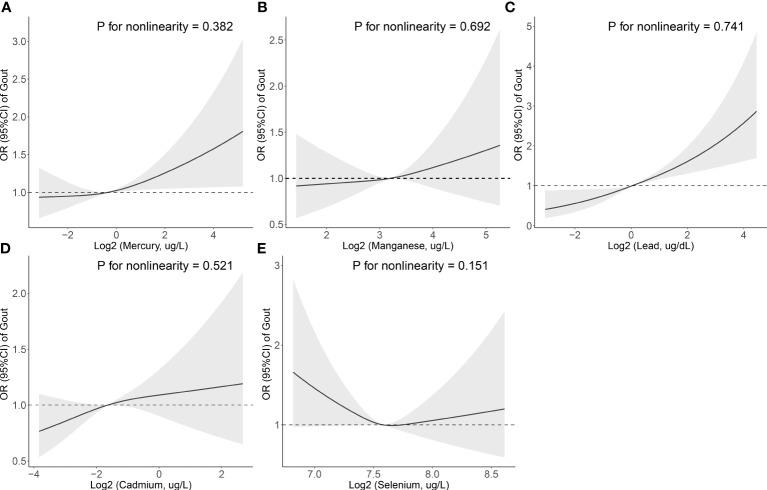
Association between the blood metal (**A**: Mercury, **B**: Manganese, **C**: Lead, **D**: Cadmium, and **E**: Selenium) levels and the prevalence of gout. The odds ratio of gout from a restricted cubic spline logistic regression model with knots at the 10^th^, 50^th^, and 90^th^ percentiles. Model was adjusted for age, sex, education level, race, poverty, smoker, alcohol user, energy intake levels, sedentary time, BMI, total cholesterol, high-density lipoprotein cholesterol, eGFR, urinary creatinine, urinary albumin, diuretics, beta-blockers, diabetes, and hypertension.

The multiple logistic regression study of the association between blood metals and the prevalence of gout is shown in **Table 4**. Participants in the 4^th^ quartile of mercury showed a statistically significant increase in the prevalence of gout (OR [95% CI]: 1.390 [1.104-1.751]), and those in the 4^th^ quartile of lead showed a statistically significant increase in the prevalence of gout (OR [95% CI]: 1.905 [1.412-2.572]) compared to the lowest referent quartile. Additionally, the restricted cubic spline demonstrated a positive linear correlation between two blood metals (mercury and lead) and the prevalence of hyperuricemia (*P* for nonlinearity = 0.382 and 0.741) (**Figure 2**).

**Table 4:** Multiple logistic regression associations of blood metal levels with the prevalence of gout in adults.

Metals	Continuous log2-transformed Metals	Quartile 1	Quartile 2	Quartile 3	Quartile 4	*P* for trend
		OR	OR (95%CI)	OR (95%CI)	OR (95%CI)	
Mercury, ug/L	1.08 (1.02-1.14) ^*^	1.00 [Reference]	1.08 (0.86-1.36)	1.11 (0.88-1.41)	1.39 (1.10-1.75) ^**^	0.029
Manganese, ug/L	1.10 (0.93-1.29)	1.00 [Reference]	1.18 (0.83-1.27)	1.01 (0.81-1.26)	1.08 (0.86-1.37)	0.917
Lead, ug/dL	1.29 (1.17-1.41) ^***^	1.00 [Reference]	1.30 (0.88-1.60)	1.13 (0.83-1.53)	1.91 (1.41-2.57) ^***^	<0.001
Cadmium, ug/L	1.08 (0.99-1.18)	1.00 [Reference]	1.21 (0.96-1.54)	1.10 (0.86-1.41)	1.10 (0.83-1.45)	0.454
Selenium, ug/L	0.79 (0.53-1.17)	1.00 [Reference]	0.79 (0.63-0.98) ^*^	0.83 (0.67-1.03)	0.85 (0.68-1.05)	0.147

Model was adjusted as age, sex, education level, race, poverty, smoker, alcohol user, energy intake levels, sedentary time, BMI, total cholesterol, high-density lipoprotein cholesterol, eGFR, urinary creatinine, urinary albumin, diuretics, beta-blockers, diabetes, and hypertension.

CI, confidence interval; ^*^ p < 0.05, ^**^ p < 0.01 and ^***^ p < 0.001.

### Mixed effects of five blood metals

**Figure 3:**
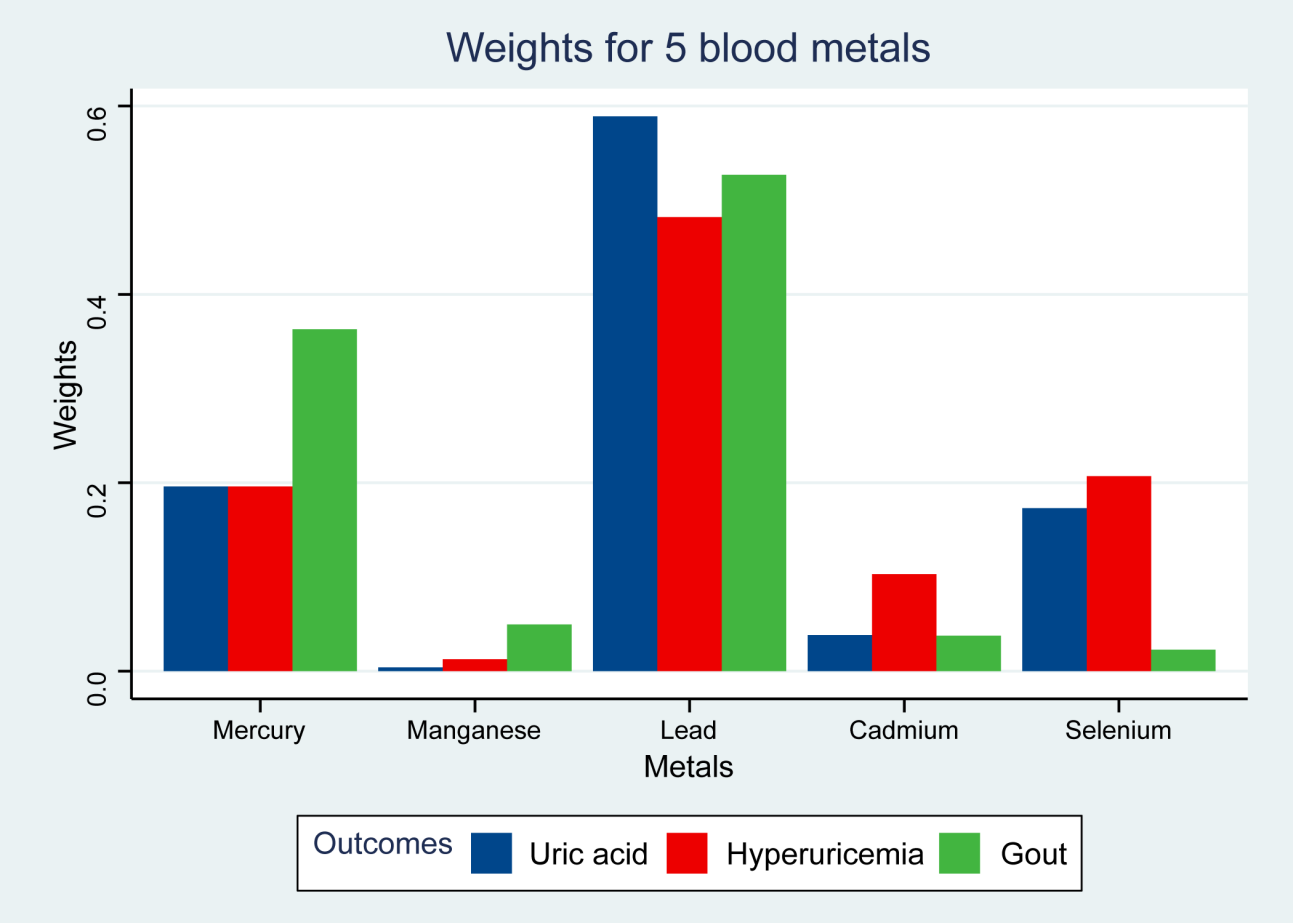
Weights from weighted quantile sum regression (WQS) for the blood metal mixtures and gout-related outcomes. Adverse model was adjusted for age, sex, education level, race, poverty, smoker, alcohol user, energy intake levels, sedentary time, BMI, total cholesterol, high-density lipoprotein cholesterol, eGFR, urinary creatinine, urinary albumin, diuretics, beta-blockers, diabetes, and hypertension.

The adverse relationships between metal mixtures and SUA or the prevalence of hyperuricemia and gout were analyzed using WQS regression analysis (**Table 5**). The WQS index of the blood metals was independently correlated with SUA (β [95%CI]: 0.17 [0.13-0.20]), hyperuricemia (OR [95%CI]: 1.29 [1.16-1.42]) and gout (OR [95%CI]: 1.35 [1.15-1.58]). After all covariates were adjusted (**Figure 3**), lead was the top predominant metal among the blood metals that were positively correlated with SUA (weight = 0.589), hyperuricemia (weight = 0.482) and gout (weight = 0.527).

**Table 5:** WQS regression model to assess the adverse association of the mixture of blood metals with gout-related outcomes.

Gout-related outcomes	β/OR	95%CI	*P* value
Uric acid	0.17	0.13-0.20	<0.001
Hyperuricemia	1.29	1.16-1.42	<0.001
Gout	1.35	1.15-1.58	<0.001

WQS regression model was adjusted as age, sex, education level, race, poverty, smoker, alcohol user, energy intake levels, sedentary time, BMI, total cholesterol, high-density lipoprotein cholesterol, eGFR, urinary creatinine, urinary albumin, diuretics, anti-gout medications, beta-blockers, diabetes, and hypertension.

OR, odds ratio; CI, confidence interval; WQS, weighted quantile sum.

### Subgroup analysis

Stratified analysis was performed to explore further whether subgroups in age (≤50 years; n=7534, and >50 years; n=7337) and sex (male; n=7328, and female; n=7543) had differential effects on the association of exposure to blood metals with the prevalence of hyperuricemia and gout (**Tables S2**, **S3**). Interestingly, sex was likely a potential modifier for the association between blood metal and the prevalence of hyperuricemia because the risk estimates for mercury (*P* for interaction = 0.001), lead (*P* for interaction < 0.001), and selenium (*P* for interaction = 0.003) in female are statistically different from those in male. In addition, compared to participants aged <50 years, a significantly stronger positive association was observed between cadmium exposure and the prevalence of hyperuricemia in those aged >50 years (*P* for interaction < 0.001). Further stratified analyses showed that age and sex did not significantly affect the modification of the association between metal exposure and the prevalence of gout (all *P* for interaction > 0.05).

## Discussion

This study analyzed the cross-sectional data from the 2011–2018 NHANES project to explore the association between five blood metals (mercury, manganese, lead, cadmium and selenium) and gout-related outcomes (including SUA, hyperuricemia and gout) in 14871 general adults. We found a positive association of an increased level of mercury, lead and selenium with gout-related outcomes. The WQS regression model showed that blood metal mixtures were associated with an increased prevalence of hyperuricemia and gout, with the greatest effect coming from lead. In addition, restricted cubic spline regression showed a linear association of blood lead level with the prevalence of hyperuricemia and gout.

Metallic mercury, inorganic mercury compounds, methylmercury and other organic compounds are the primary forms of mercury (14). Mercury exposure occurs mostly through the consumption of methylmercury-contaminated fish and shellfish, the inhalation of mercury vapor, and the use of mercury-containing items (15). Mercury is a recognized toxic substance, whose effect on hyperuricemia is controversial. Park et al. found a positive association between the level of blood mercury and SUA among women. However, there was no statistical association between the fourth quartile grouping of blood mercury and hyperuricemia (16). Jung et al. reported no significant association between the level of mercury in blood and SUA or hyperuricemia in their study (12), which differed from the results of our study, where a positive association was found between the level of mercury in blood and SUA. Meanwhile, the level of mercury in blood was associated with an increased prevalence of hyperuricemia and gout. The different results may be due to geographical and ethnic variability. In a fundamental study, it was discovered that oral mercuric chloride might enhance the amount of SUA in rabbits. However, the specific mechanism required further investigation (17).

Most lead comes from human activities, including burning fossil fuels, mining, smelting and exposure to products containing lead compound (18). Lead is a widespread poison, and long-term exposure to a high level of it can lead to the occurrence of many chronic diseases (19). Jung et al. showed a positive correlation between the level of lead in blood and SUA. However, this association was only among women, and no association was found between the level of lead in blood and the prevalence of hyperuricemia (12). Shadick et al. reported that chronic lead accumulation was positively correlated with SUA among older adults but not with hyperuricemia or gout (20). Dai et al. found that continuous exposure to lead had an independent effect on SUA among both men and women. In contrast, exposure to lead was significantly associated with hyperuricemia among women but not men (21). Krishnan et al. found that the level of lead in blood was associated with an increased prevalence of hyperuricemia and gout, which was consistent with our findings (22). However, a negative association between urinary lead and SUA was found in a cross-sectional study on Chinese traffic police (23). Buser et al. showed a positive correlation between urinary lead and glomerular filtration rate as well as a negative correlation between blood lead and glomerular filtration rate (24). This finding seems to be able to explain the different associations of blood and urinary lead concentration with SUA.

Selenium is commonly found in organic or inorganic forms in living organisms (25), which is necessary to maintain normal immune system functions. However, a high selenium intake can also be somewhat toxic (26). A negative association between serum selenium level and SUA was found in a 2-year longitudinal study on patients undergoing haemodialysis (27). Wang et al. found a positive linear association between plasma selenium concentration and the prevalence of hyperuricemia, but this association was diminished when a polymetallic model was utilized (28). It was found in an intervention study that dietary selenium intake was positively associated with the level of SUA in early and mid-pregnancy (29). In our study, exposure to selenium was associated with an elevated SUA and incidence of hyperuricemia but not with gout incidence. This disparity could be due to characteristics such as race, age, and occupation.

Cadmium is distributed in the environment at a low level, including cadmium fumes and cadmium compounds (30), the primary uses of which are in manufacturing batteries, pigments and electronic products (31). Zeng et al. explored the relationship between the level of blood cadmium with SUA and hyperuricemia. The regression analysis showed that the level of cadmium in blood was associated with an increased prevalence of hyperuricemia, but this association only existed among women. A non-linear relationship was found between the level of cadmium in blood and hyperuricemia among men, and this sex difference might be a hormonal effect (13). Sun et al. found that the positive correlation between the level of blood cadmium and SUA only existed in men (11). Zhang et al. found that cadmium in blood and urine was a risk factor for gout flares (32). However, none of these associations was found in our study.

Inconsistent conclusions have been reached for studies on the association of metals with hyperuricemia and gout, which may be related to differences in demographic characteristics and the interference of confounding factors. In addition to exploring the relationship between a single metal and SUA, researchers have increasingly focused on the effect of metal mixtures on it. Ma et al. investigated the combined effect of metal mixtures in urine on SUA and hyperuricemia using three statistical models, through all of which significant roles of arsenic, cadmium and cobalt were found in changes in SUA and hyperuricemia (33). Using Bayesian kernel machine regression (BKMR) analysis, Gao et al. found that exposure to metal mixtures was associated with a higher level of SUA, with the most considerable effect coming from blood lead, urinary cadmium and urinary arsenic (in males only) (34). Through WQS analysis, we found that blood metal mixtures were associated with an increased prevalence of hyperuricemia and gout, with the enormous effect coming from lead.

The mechanisms underlying the effect of lead level in the human body on SUA are unclear. It is well known that hyperuricemia, gout, and kidney diseases are closely related (35). Studies have shown that starting with glomerular and tubulointerstitial changes, a long-term low-level exposure to lead can lead to irreversible functional and morphological changes in kidneys, which ultimately leads to renal failure, hypertension and hyperuricemia (36). It has been reported that oxidative stress is one mechanism of lead-induced toxicity, mainly by inducing ROS production or depleting antioxidant reserves (37). Kasperczyk et al. demonstrated that occupational exposure to lead could induce xanthine oxidase activity and increase the level of SUA (38). In addition, lead can not only interfere with purine metabolism in the human body (39) but also induce inflammatory responses (40). In conclusion, the association mechanism between lead and SUA is complex, and more research is needed for exploration.

The main advantage of this study is that it uses the WQS regression to explore the mixture effects of environmental pollutants on gout-related outcomes, which is more in line with the reality of exposure. In terms of study design, most of them involved single metal exposure. However, humans are simultaneously exposed to multiple metals, of which the levels are usually highly correlated. The WQS statistical approach ensures that our results are not confounded by exposure collinearity issues, which allows a specific metal’s statistical significance to be independent of the effect of co-exposure to other metals. Moreover, we identified that lead was the largest contributor to the positive association of metal mixtures with the prevalence of hyperuricemia and gout, which had rarely been assessed in previous studies. Thirdly, since the sample size of this study is large and representative, the conclusions can be generalized to other American adults. Fourthly, the exposure to metals in blood is analyzed in this study, which is different from that in urine (41). Akerstrom et al. found that blood cadmium was a valid biomarker of exposure to cadmium, while urinary cadmium was susceptible to blood cadmium and urinary protein (42). Finally, the most known confounding factors are adjusted in our study.

This study has several limitations. First of all, as a cross-sectional study, we were unable to demonstrate a causal association. Secondly, a history of gout based on self-reports might not be completely accurate. Thirdly, blood metals were measured simultaneously without repeated measurements, which might not represent long-term exposure to metals. Finally, the exact mechanism through which metals caused changes in SUA was not known and needed to be confirmed by further research.

## Conclusion

In this cross-sectional study based on general adults, we found an association between the level of blood mercury, lead and selenium and SUA. Our findings suggested that metal mixtures in blood were associated with an increased prevalence of hyperuricemia and gout, with the greatest effect coming from lead. Additional research is necessary to confirm these findings and clarify the underlying mechanism.

## Data availability statement

The raw data supporting the conclusions of this article will be made available by the authors, without undue reservation.

## Ethics statement

The studies involving human participants were reviewed and approved by the National Center for Health Statistics Research Ethics Review Board. The patients/participants provided their written informed consent to participate in this study.

## Author contributions

JX: Conceptualization, Methodology, Formal analysis, Writing-original draft. XZ: Conceptualization, Methodology, Software, Visualization, Writing-original draft. RH: Project administration, Supervision. YX: Project administration, Supervision. SS: Conceptualization, Methodology, Supervision. YZ: Conceptualization, Project administration, Supervision. LZ: Conceptualization, Methodology, Project administration, Writing-review and editing, Supervision. All authors contributed to the article and approved the submitted version.

## Funding

This work was supported by the Science and Technology Talents Support Program of Shaanxi Provincial People’s Hospital (No. 2021BJ-10, No. 2021BJ-06).

## Acknowledgments

We appreciate the people who contributed to the NHANES data we studied.

## Conflict of interest

The authors declare that the research was conducted in the absence of any commercial or financial relationships that could be construed as a potential conflict of interest.

## Publisher’s note

All claims expressed in this article are solely those of the authors and do not necessarily represent those of their affiliated organizations, or those of the publisher, the editors and the reviewers. Any product that may be evaluated in this article, or claim that may be made by its manufacturer, is not guaranteed or endorsed by the publisher.

## References

[1] So A Thorens B. Uric acid transport and disease. J Clin Invest (2010) 120(6):1791–9. doi: 10.1172/JCI42344 PMC2877959 20516647

[2] Maiuolo J Oppedisano F Gratteri S Muscoli C Mollace V. Regulation of uric acid metabolism and excretion. Int J Cardiol (2016) 213:8–14. doi: 10.1016/j.ijcard.2015.08.109 26316329

[3] Fenando A Rednam M Gujarathi R Widrich J. Gout. In: StatPearls. Treasure Island (FL: StatPearls Publishing (2022). 31536213

[4] Richette P Doherty M Pascual E Barskova V Becce F Castaneda J. 2018 updated European league against rheumatism evidence-based recommendations for the diagnosis of gout. Ann Rheum Dis (2020) 79(1):31–8. doi: 10.1136/annrheumdis-2019-215315 31167758

[5] Madero M Sarnak MJ Wang X Greene T Beck GJ Kusek JW. Uric acid and long-term outcomes in CKD. Am J Kidney Dis (2009) 53(5):796–803. doi: 10.1053/j.ajkd.2008.12.021 19303683 PMC2691553

[6] Kanbay M Segal M Afsar B Kang DH Rodriguez-Iturbe B Johnson RJ. The role of uric acid in the pathogenesis of human cardiovascular disease. Heart. (2013) 99(11):759–66. doi: 10.1136/heartjnl-2012-302535 23343689

[7] Tseng WC Chen YT Ou SM Shih CJ. Tarng DC. U-shaped association between serum uric acid levels with cardiovascular and all-cause mortality in the elderly: The role of malnourishment. J Am Heart Assoc (2018) 7(4):e007523. doi: 10.1161/JAHA.117.007523 29440009 PMC5850189

[8] Dehghan A van Hoek M Sijbrands EJ Hofman A Witteman JC. High serum uric acid as a novel risk factor for type 2 diabetes. Diabetes Care (2008) 31(2):361–2. doi: 10.2337/dc07-1276 17977935

[9] de Oliveira EP Burini RC. High plasma uric acid concentration: causes and consequences. Diabetol Metab Syndr (2012) 4:12. doi: 10.1186/1758-5996-4-12 22475652 PMC3359272

[10] Rehman K Fatima F Waheed I Akash M. Prevalence of exposure of heavy metals and their impact on health consequences. J Cell Biochem (2018) 119(1):157–84. doi: 10.1002/jcb.26234 28643849

[11] Sun H Wang N Chen C Nie X Han B Li Q. Cadmium exposure and its association with serum uric acid and hyperuricemia. Sci Rep (2017) 7(1):550. doi: 10.1038/s41598-017-00661-3 28373703 PMC5428845

[12] Jung W Kim Y Lihm H Kang J. Associations between blood lead, cadmium, and mercury levels with hyperuricemia in the Korean general population: A retrospective analysis of population-based nationally representative data. Int J Rheum Dis (2019) 22(8):1435–44. doi: 10.1111/1756-185X.13632 31215160

[13] Zeng A Li S Zhou Y Sun D. Association between low-level blood cadmium exposure and hyperuricemia in the American general population: a cross-sectional study. Biol Trace Elem Res (2022) 200(2):560–7. doi: 10.1007/s12011-021-02700-7 33837913

[14] Bjorklund G Dadar M Mutter J Aaseth J. The toxicology of mercury: Current research and emerging trends. Environ Res (2017) 159:545–54. doi: 10.1016/j.envres.2017.08.051 28889024

[15] Ferreira-Rodriguez N Castro AJ Tweedy BN Quintas-Soriano C Vaughn CC. Mercury consumption and human health: Linking pollution and social risk perception in the southeastern united states. J Environ Manage (2021) 282:111528. doi: 10.1016/j.jenvman.2020.111528 33172704

[16] Park J Kim Y. Associations of blood heavy metals with uric acid in the Korean general population: Analysis of data from the 2016-2017 Korean national health and nutrition examination survey. Biol Trace Elem Res (2021) 199(1):102–12. doi: 10.1007/s12011-020-02152-5 32342340

[17] Ali S Hussain S Khan R Mumtaz S Ashraf N Andleeb S. Renal toxicity of heavy metals (cadmium and mercury) and their amelioration with ascorbic acid in rabbits. Environ Sci Pollut Res Int (2019) 26(4):3909–20. doi: 10.1007/s11356-018-3819-8 30547340

[18] Ravipati ES Mahajan NN Sharma S Hatware KV Patil K. The toxicological effects of lead and its analytical trends: an update from 2000 to 2018. Crit Rev Anal Chem (2021) 51(1):87–102. doi: 10.1080/10408347.2019.1678381 31650860

[19] Hernberg S. Lead poisoning in a historical perspective. Am J Ind Med (2000) 38(3):244–54. doi: 10.1002/1097-0274(200009)38:3<244::AID-AJIM3>3.0.CO;2-F 10940962

[20] Shadick NA Kim R Weiss S Liang MH Sparrow D Hu H. Effect of low level lead exposure on hyperuricemia and gout among middle aged and elderly men: the normative aging study. J Rheumatol (2000) 27(7):1708–12. 10914856

[21] Dai H Huang Z Deng Q Li Y Xiao T Ning X. The effects of lead exposure on serum uric acid and hyperuricemia in Chinese adults: A cross-sectional study. Int J Environ Res Public Health (2015) 12(8):9672–82. doi: 10.3390/ijerph120809672 PMC4555305 26295243

[22] Krishnan E Lingala B Bhalla V. Low-level lead exposure and the prevalence of gout: an observational study. Ann Intern Med (2012) 157(4):233–41. doi: 10.7326/0003-4819-157-4-201208210-00003 22910934

[23] Dai X Deng Q Guo D Ni L Li J Chen Z. Association of urinary metal profiles with serum uric acid: a cross-sectional study of traffic policemen in wuhan, China. BMJ Open (2019) 9(5):e022542. doi: 10.1136/bmjopen-2018-022542 PMC6530447 31079077

[24] Buser MC Ingber SZ Raines N Fowler DA Scinicariello F. Urinary and blood cadmium and lead and kidney function: NHANES 2007-2012. Int J Hyg Environ Health (2016) 219(3):261–7. doi: 10.1016/j.ijheh.2016.01.005 PMC5685486 26852280

[25] Lenz M Lens PN. The essential toxin: the changing perception of selenium in environmental sciences. Sci Total Environ (2009) 407(12):3620–33. doi: 10.1016/j.scitotenv.2008.07.056 18817944

[26] Garcia-Barrera T. Selenium and mercury: Their interactions and roles in living organisms. In: Nriagu JO Skaar EP Eds Trace Metals and Infectious Diseases [Internet] Cambridge (MA): MIT Press Chapter 14. (2015). 33886186

[27] Marti DML Agil A Navarro-Alarcon M Lopez-Ga DLSH Palomares-Bayo M Oliveras-Lopez MJ. Altered serum selenium and uric acid levels and dyslipidemia in hemodialysis patients could be associated with enhanced cardiovascular risk. Biol Trace Elem Res (2011) 144(1-3):496–503. doi: 10.1007/s12011-011-9152-4 21789541

[28] Wang T Lv Z Wen Y Zou X Zhou G Cheng J. Associations of plasma multiple metals with risk of hyperuricemia: A cross-sectional study in a mid-aged and older population of China. Chemosphere (2022) 287(Pt 3):132305. doi: 10.1016/j.chemosphere.2021.132305 34563770

[29] Pieczynska J Placzkowska S Sozanski R Orywal K Mroczko B Grajeta H. Is maternal dietary selenium intake related to antioxidant status and the occurrence of pregnancy complications? J Trace Elem Med Biol (2019) 54:110–7. doi: 10.1016/j.jtemb.2019.04.010 31109600

[30] Kubier A Wilkin RT Pichler T. Cadmium in soils and groundwater: A review. Appl Geochem (2019) 108:1–16. doi: 10.1016/j.apgeochem.2019.104388 32280158 PMC7147761

[31] Jarup L Akesson A. Current status of cadmium as an environmental health problem. Toxicol Appl Pharmacol (2009) 238(3):201–8. doi: 10.1016/j.taap.2009.04.020 19409405

[32] Zhang H Li H Green AP Wang M Yan F Li M. Association of low-level environmental exposure to cadmium and lead with gout flare using a cohort study design. Chemosphere (2021) 280:130648. doi: 10.1016/j.chemosphere.2021.130648 33932909

[33] Ma Y Hu Q Yang D Zhao Y Bai J Mubarik S. Combined exposure to multiple metals on serum uric acid in NHANES under three statistical models. Chemosphere (2022) 301:134416. doi: 10.1016/j.chemosphere.2022.134416 35490746

[34] Gao W Tong L Zhao S Sun M Fang J Xu Y. Exposure to cadmium, lead, mercury, and arsenic and uric acid levels: Results from NHANES 2007-2016. Biol Trace Elem Res (2022). doi: 10.1007/s12011-022-03309-0 35809185

[35] Roughley M Sultan AA Clarson L Muller S Whittle R Belcher J. Risk of chronic kidney disease in patients with gout and the impact of urate lowering therapy: a population-based cohort study. Arthritis Res Ther (2018) 20(1):243. doi: 10.1186/s13075-018-1746-1 30376864 PMC6235219

[36] Blazar BA Rodrick ML O'Mahony JB Wood JJ Bessey PQ Wilmore DW. Suppression of natural killer-cell function in humans following thermal and traumatic injury. J Clin Immunol (1986) 6(1):26–36. doi: 10.1007/BF00915361 3485653

[37] Matovic V Buha A Ethukic-Cosic D Bulat Z. Insight into the oxidative stress induced by lead and/or cadmium in blood, liver and kidneys. Food Chem Toxicol (2015) 78:130–40. doi: 10.1016/j.fct.2015.02.011 25681546

[38] Kasperczyk S Dobrakowski M Ostalowska A Kasperczyk A Wilczyinski S Wyparlo-Wszelaki M. Lead-elevated activity of xanthine oxidase in lead-exposed workers. Med Pr (2013) 64(2):175–80. doi: 10.13075/mp.5893/2013/0013 23829061

[39] Kilikdar D Mukherjee D Mitra E Ghosh AK Basu A Chandra AM. Protective effect of aqueous garlic extract against lead-induced hepatic injury in rats. Indian J Exp Biol (2011) 49(7):498–510. 21800501

[40] Boskabady M Marefati N Farkhondeh T Shakeri F Farshbaf A Boskabady MH. The effect of environmental lead exposure on human health and the contribution of inflammatory mechanisms, a review. Environ Int (2018) 120:404–20. doi: 10.1016/j.envint.2018.08.013 30125858

[41] Kuo CC Weaver V Fadrowski JJ Lin YS Guallar E Navas-Acien A. Arsenic exposure, hyperuricemia, and gout in US adults. Environ Int (2015) 76:32–40. doi: 10.1016/j.envint.2014.11.015 25499256

[42] Akerstrom M Barregard L Lundh T Sallsten G. The relationship between cadmium in kidney and cadmium in urine and blood in an environmentally exposed population. Toxicol Appl Pharmacol (2013) 268(3):286–93. doi: 10.1016/j.taap.2013.02.009 23454399

