# Supplementary material for: Associations of metal exposure with hyperuricemia and gout in general adults
Source: Front Endocrinol (Lausanne). 2022 Dec 2;13:1052784. doi: 10.3389/fendo.2022.1052784 (PMC9755211; doi:10.3389/fendo.2022.1052784)
Supplement: Supplementary file 1 [file DataSheet_1.pdf]

Online Supplementary Material

Associations of Metal Exposure with Hyperuricemia and Gout in General Adults

Jing Xu; Xu Zhu; Rutai Hui; Yujie Xing; Junkui Wang; Shuang Shi; Yong Zhang; Ling Zhu

Figure S1. Eligible participants in the evaluation of the influence between blood metals and serum uric acid (SUA), hyperuricemia or gout in the general adult population.

Table S1. Concentrations and detection rate of the blood heavy metals.

Figure S2. Pairwise spearman correlation coefficients among blood heavy metals in the general population.

Figure S3. Histogram of distribution of residuals of linear regression investigating the associations of blood metal levels with serum uric acid.

Table S2. Subgroup analysis based on different age (≤50 years; n=7534, and >50 years; n=7337) and gender (male; n=7328, and female; n=7543) for the associations of the quartiles of blood metal levels with hyperuricemia by logistic regression.

Table S3. Subgroup analysis based on different age (≤50 years; n=7534, and >50 years; n=7337) and sex (male; n=7328, and female; n=7543) for the associations of the quartiles of blood metal levels with gout by logistic regression.

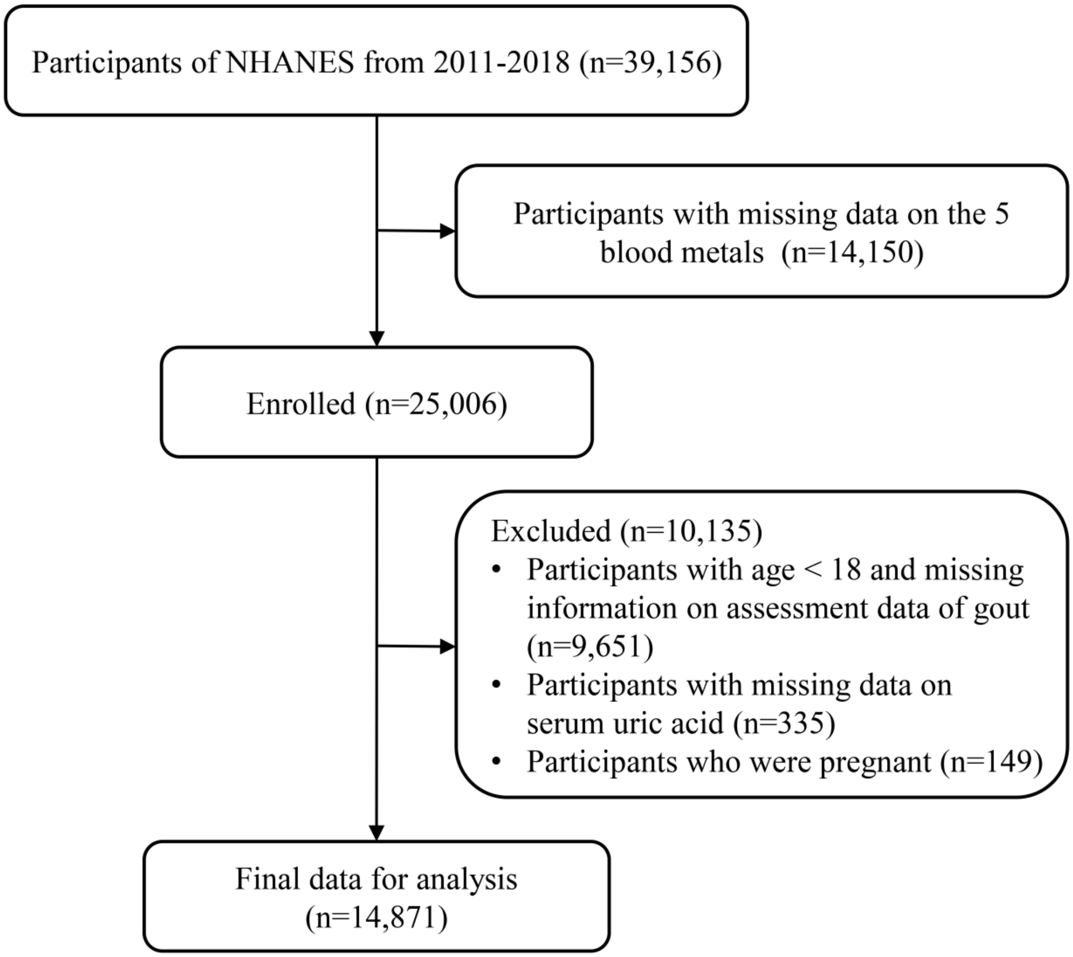

Figure S1. Eligible participants in the evaluation of the influence between blood metals and serum uric acid (SUA), hyperuricemia or gout in the general adult population.

Table S1. Concentrations and detection rate of the blood heavy metals.

Metals	N	LODmax	≥LODmax(%)[a]	5th	25th	50th	75th	95th
Mercury, ug/L	14871	0.28	100.0	0.20	0.40	0.78	1.66	5.63
Manganese, ug/L	14871	0.99	100.0	5.34	7.41	9.26	11.66	16.69
Lead, ug/dL	14871	0.07	99.0	0.35	0.64	1.02	1.63	3.32
Cadmium, ug/L	14871	0.10	100.0	0.11	0.19	0.32	0.58	1.53
Selenium, ug/L	14871	24.48	100.0	156.10	176.86	191.51	207.20	236.15

N, number of urinary samples; LOD, limit of detection; 5th, 5th percentile; 25th, 25th percentile; 50th, 50th percentile; 75th, 75th percentile; 95th, 95th percentile;

[a] Percentage of metabolite concentrations at or above the maximum limit of detection (< LODmax). All concentrations below the LODmax (< LODmax) were substituted with a value of LODmax divided by square root of two (√2).

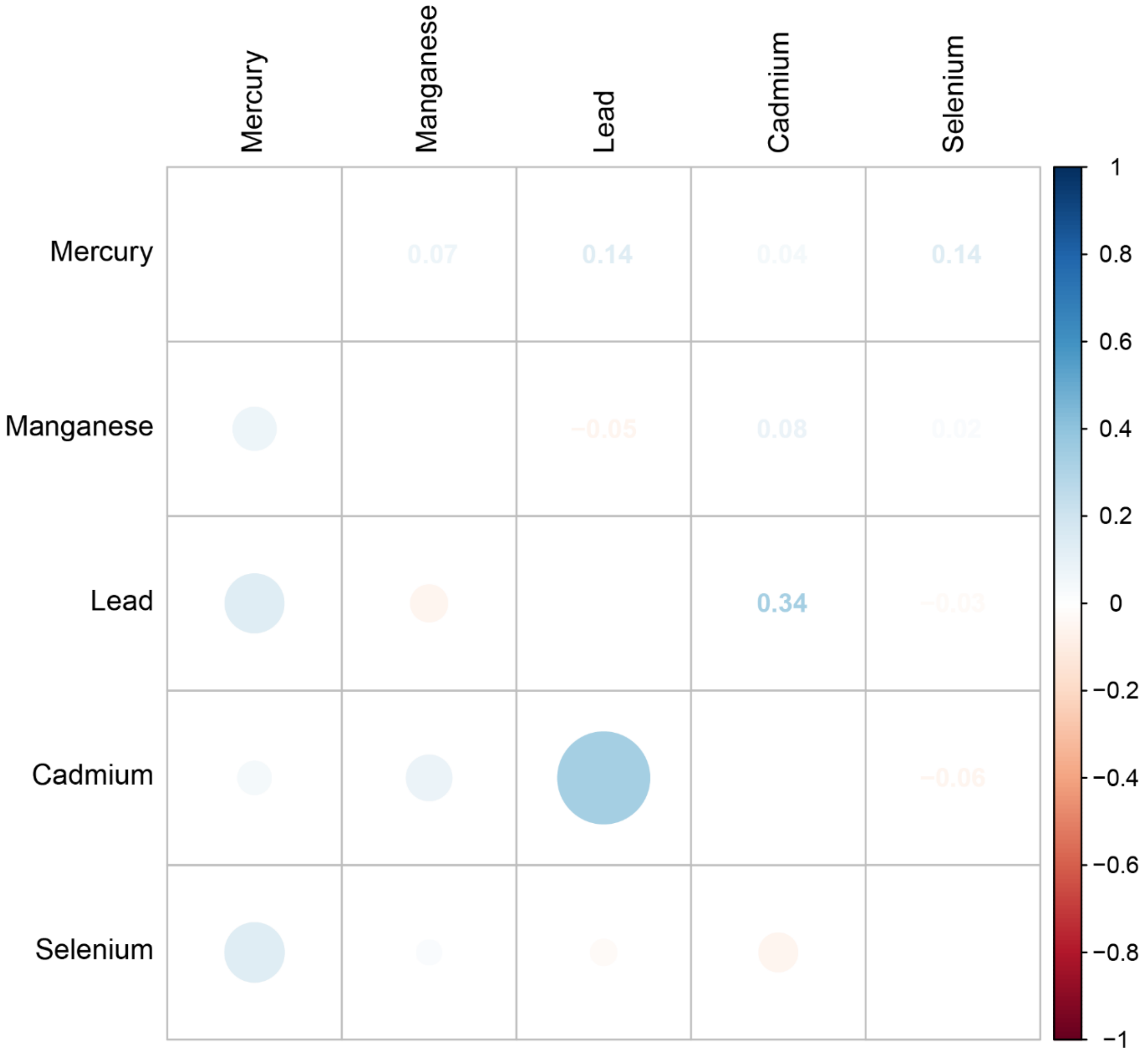

Figure S2. Pairwise spearman correlation coefficients among blood metals in the general population.

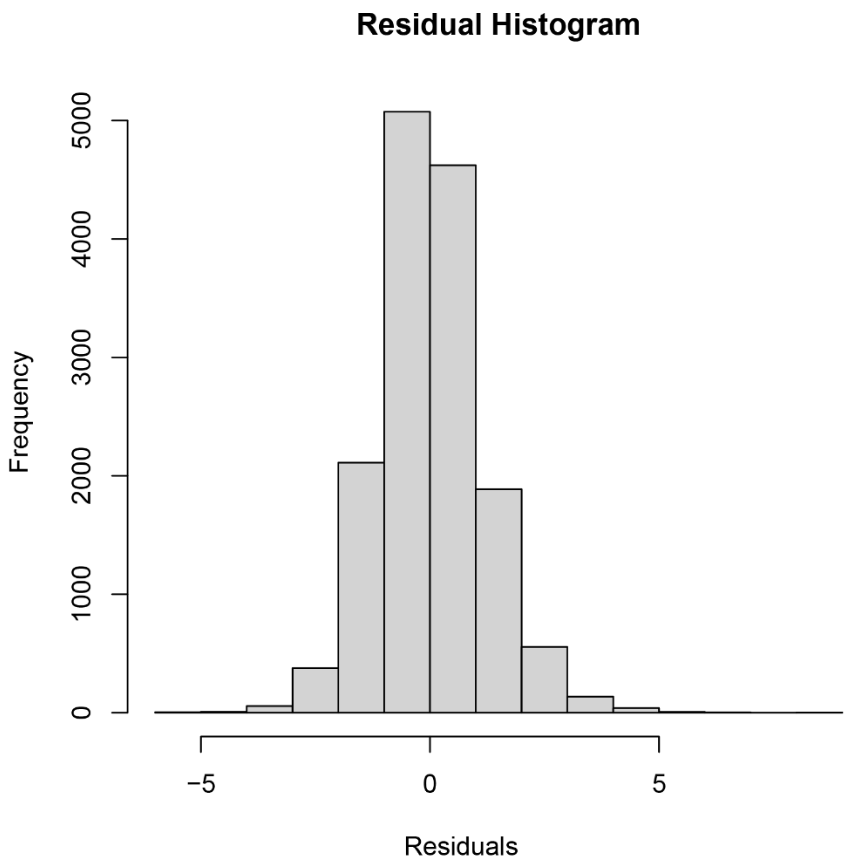

Figure S3. Histogram of distribution of residuals of linear regression investigating the associations of blood metal levels with serum uric acid. The model included age, sex, education level, race, poverty, smoker, alcohol user, energy intake levels, sedentary time, BMI, total cholesterol, high-density lipoprotein cholesterol, eGFR, urinary creatinine, urinary albumin, diuretics, anti-gout medications, beta-blockers, diabetes, and hypertension.

Table S2. Subgroup analysis based on different age (≤50 years; n=7534, and >50 years; n=7337) and sex (male; n=7328, and female; n=7543) for the associations of the quartiles of blood metal levels with hyperuricemia by logistic regression.

	Quartile 1	Quartile 2	Quartile 3	Quartile 4	*p-t*	*p-int*
	β	β (95%CI)	β (95%CI)	β (95%CI)		
Mercury, µg/L						
≤50 years	1.00 [Reference]	1.213 (1.008-1.461)	0.941 (0.769-1.152)	1.136 (0.921-1.401)	0.045	0.258
>50 years	1.00 [Reference]	1.201 (1.002-1.439)	1.141 (0.949-1.371)	1.399 (1.161-1.684)	0.004	
Male	1.00 [Reference]	1.133 (0.955-1.345)	.888 (0.741-1.064)	1.108 (0.925-1.327)	0.028	0.001
Female	1.00 [Reference]	1.315 (1.077-1.606)	1.293 (1.053-1.586)	1.606 (1.297-1.989)	<0.001	
Manganese, µg/L						
≤50 years	1.00 [Reference]	1.006 (0.821-1.233)	1.013 (0.824-1.244)	1.208 (0.978-1.491)	0.188	0.868
>50 years	1.00 [Reference]	0.909 (0.770-1.073)	0.919 (0.775-1.090)	0.899 (0.747-1.082)	0.605	
Male	1.00 [Reference]	0.893 (0.757-1.052)	0.958 (0.808-1.136)	1.100 (0.913-1.324)	0.164	0.072
Female	1.00 [Reference]	0.964 (0.785-1.184)	0.898 (0.731-1.102)	0.951 (0.771-1.173)	0.771	
Lead, µg/dL						
≤50 years	1.00 [Reference]	1.053 (0.879-1.263)	1.087 (0.884-1.337)	1.413 (1.130-1.768)	0.019	0.503
>50 years	1.00 [Reference]	1.168 (0.914-1.492)	1.210 (0.954-1.536)	1.408 (1.104-1.797)	0.023	
Male	1.00 [Reference]	1.029 (0.842-1.258)	1.205 (0.982-1.479)	1.489 (1.205-1.841)	<0.001	<0.001
Female	1.00 [Reference]	1.168 (0.953-1.431)	1.148 (0.925-1.425)	1.520 (1.204-1.918)	0.004	
Cadmium, µg/L						
≤50 years	1.00 [Reference]	1.067 (0.892-1.276)	0.855 (0.682-1.073)	0.821 (0.633-1.064)	0.145	<0.001
>50 years	1.00 [Reference]	1.180 (0.969-1.435)	1.134 (0.932-1.380)	1.395 (1.115-1.745)	0.026	
Male	1.00 [Reference]	1.146 (0.972-1.350)	1.088 (0.899-1.318)	1.166 (0.934-1.455)	0.357	0.155
Female	1.00 [Reference]	1.048 (0.842-1.306)	0.962 (0.770-1.202)	1.182 (0.912-1.530)	0.267	
Selenium, µg/L						
≤50 years	1.00 [Reference]	1.171 (0.951-1.443)	1.065 (0.864-1.311)	1.292 (1.052-1.586)	0.064	0.369
>50 years	1.00 [Reference]	1.172 (0.986-1.394)	1.112 (0.931-1.328)	1.292 (1.083-1.541)	0.037	
Male	1.00 [Reference]	1.056 (0.882-1.264)	0.825 (0.687-0.991)	1.016 (0.850-1.213)	0.027	0.003
Female	1.00 [Reference]	1.227 (1.007-1.494)	1.385 (1.135-1.690)	1.602 (1.310-1.959)	<0.001	

Model was adjusted as age, sex, education level, race, poverty, smoker, alcohol user, energy intake levels, sedentary time, BMI, total cholesterol, high-density lipoprotein cholesterol, eGFR, urinary creatinine, urinary albumin, diuretics, anti-gout medications, beta-blockers, diabetes, and hypertension when they were not the strata variables. *p-t, p* for trend; *p-int*, p for interaction.

Table S3. Subgroup analysis based on different age (≤50 years; n=7534, and >50 years; n=7337) and gender (male; n=7328, and female; n=7543) for the associations of the quartiles of blood metal levels with gout by logistic regression.

	Quartile 1	Quartile 2	Quartile 3	Quartile 4	*p-t*	*p-int*
	β	β (95%CI)	β (95%CI)	β (95%CI)		
Mercury, μg/L						
≤50 years	1.00 [Reference]	0.886 (0.507-1.549)	1.250 (0.725-2.154)	1.509 (0.880-2.588)	0.250	0.534
>50 years	1.00 [Reference]	1.113 (0.865-1.433)	1.069 (0.824-1.386)	1.337 (1.035-1.727)	0.124	
Male	1.00 [Reference]	1.097 (0.822-1.465)	1.130 (0.841-1.517)	1.508 (1.138-1.999)	0.019	0.171
Female	1.00 [Reference]	1.084 (0.742-1.584)	1.132 (0.767-1.671)	1.150 (0.758-1.742)	0.909	
Manganese, μg/L						
≤50 years	1.00 [Reference]	1.551 (0.881-2.731)	1.584 (0.901-2.784)	1.390 (0.757-2.553)	0.385	0.212
>50 years	1.00 [Reference]	0.952 (0.757-1.198)	0.907 (0.713-1.154)	1.052 (0.815-1.357)	0.713	
Male	1.00 [Reference]	1.142 (0.883-1.475)	1.172 (0.898-1.529)	1.177 (0.879-1.575)	0.589	0.108
Female	1.00 [Reference]	0.793 (0.543-1.160)	0.718 (0.487-1.058)	0.933 (0.630-1.383)	0.3116	
Lead, μg/dL						
≤50 years	1.00 [Reference]	1.135 (0.665-1.937)	1.554 (0.888-2.722)	1.999 (1.119-3.572)	0.087	0.343
>50 years	1.00 [Reference]	1.107 (0.759-1.615)	0.996 (0.688-1.443)	1.792 (1.245-2.579)	<0.001	
Male	1.00 [Reference]	1.361 (0.909-2.038)	1.041 (0.691-1.570)	1.935 (1.296-2.888)	<0.001	0.080
Female	1.00 [Reference]	0.894 (0.558-1.431)	1.283 (0.816-2.018)	1.930 (1.210-3.080)	0.001	
Cadmium, μg/L						
≤50 years	1.00 [Reference]	1.936 (1.174-3.193)	1.116 (0.564-2.206)	1.875 (0.945-3.720)	0.030	0.075
>50 years	1.00 [Reference]	1.080 (0.827-1.412)	1.117 (0.856-1.458)	1.027 (0.752-1.403)	0.828	
Male	1.00 [Reference]	1.485 (1.127-1.957)	1.232 (0.913-1.663)	1.268 (0.895-1.796)	0.046	0.054
Female	1.00 [Reference]	0.693 (0.435-1.102)	0.850 (0.549-1.317)	0.785 (0.475-1.296)	0.460	
Selenium, μg/L						
≤50 years	1.00 [Reference]	0.500 (0.268-0.936)	1.029 (0.613-1.726)	0.870 (0.513-1.476)	0.095	0.062
>50 years	1.00 [Reference]	0.826 (0.653-1.046)	0.753 (0.590-0.962)	0.802 (0.629-1.022)	0.108	
Male	1.00 [Reference]	0.838 (0.635-1.105)	0.907 (0.692-1.188)	0.832 (0.633-1.093)	0.517	0.327
Female	1.00 [Reference]	0.729 (0.504-1.054)	0.681 (0.461-1.007)	0.904 (0.623-1.314)	0.164	

Model was adjusted as age, sex, education level, race, poverty, smoker, alcohol user, energy intake levels, sedentary time, BMI, total cholesterol, high-density lipoprotein cholesterol, eGFR, urinary creatinine, urinary albumin, diuretics, beta-blockers, diabetes, and hypertension when they were not the strata variables. *p-t, p* for trend; *p-int*, p for interaction.
